# Genetic Evidence that Humans Have Pushed Orang-utans to the Brink of Extinction

**DOI:** 10.1371/journal.pbio.0040057

**Published:** 2006-01-24

**Authors:** Liza Gross

Humans' capacity to alter the environment has grown exponentially over the past century, challenging other species to adapt to a transfigured landscape. Over some 15,000 plants and animals face extinction, the overwhelming majority threatened by increasingly fragmented, polluted, and deteriorating habitats. These forces are most acute in Africa and Asia, where 25% of all primate species are threatened. Chimpanzees, bonobos, orang-utans, and gorillas could vanish from the wild completely within a matter of decades.

In Africa, deforestation, illegal hunting, and disease outbreaks are decimating chimp, bonobo, and gorilla populations. Orang-utans, the only non-African great ape, face similar threats, compounded by agricultural conversion of logged forests. But because orang-utans spend most of their time in trees and avoid open spaces, they may suffer most from deforestation. A new study by Benoit Goossens, Lounès Chikhi, Michael Bruford, and their colleagues shows that the case for orang-utans is in fact even more desperate than previously believed. Using computer simulations and the largest genetic dataset ever collected from wild orangutans, the authors build up a picture of alarming—and very recent—declines of orang-utan populations of over 95%.

Orang-utans, Malaysian for “man of the forest,” once ranged from Indonesia to southern China and northeastern India, but now struggle to survive in isolated forest patches in northern Sumatra and Borneo. Over 66% of the Sumatra orang-utan population was killed between 1993 and 2000; 33% of the Borneo population may have succumbed to drought and fire between 1996 and 1997 alone. When a 1987 study estimated that over 20,000 orang-utans persisted in Sabah, Malaysia, in the 1980s, it was challenged as too optimistic.

Developing effective conservation and recovery programs depends on determining when the decline began, its trajectory, and the original population size. To shed light on these questions, Goossens and Chikhi used three statistical methods to mine the genetic material of wild orang-utans in Eastern Sabah, scarred by over 50 years of large-scale commercial logging and agriculture.

For the genetic analysis, the authors and their local assistants collected hair from tree nests—some nearly 100 feet above the ground—and feces found under nests or near orang-utans encountered along the Kinabatangan River. Two hundred orang-utans were identified using genetic markers called microsatellites, tandem repeats of short DNA motifs that are sometimes used as genetic fingerprints. The authors used 14 microsatellites from each animal in an analysis that compares observed genetic diversity with that expected for a stable population, based on the number of alleles (gene pairs, or genetic motifs, at one chromosomal location) and expected heterozygosity (the alleles in the pair differ). [Fig pbio-0040057-g001]

**Figure pbio-0040057-g001:**
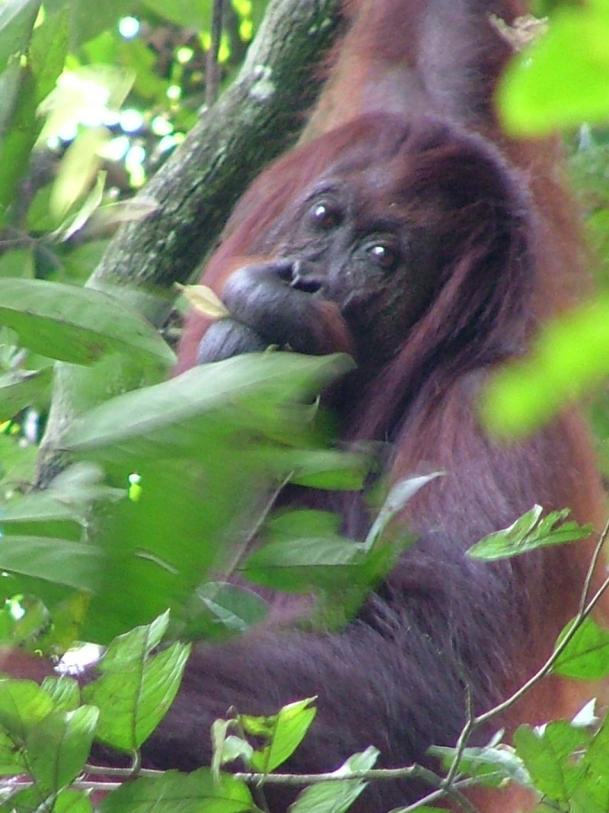
Ongoing deforestation and palm oil plantations leave no room for the orang-utan

When population size drops significantly, genetic diversity decreases as well, but in a very specific way: rare alleles are lost whereas heterozygosity remains relatively little affected. And it is this signature that the authors were looking for. The authors first simulated what the expected heterozygosity would be, based on the number of alleles and sample size (nine forest blocks were sampled) for each population and microsatellite locus, and compared these values to those seen in the collected genetic data. Three different mutation models were used to simulate variations of microsatellite repeat number under different mutational processes and demographic histories to detect evidence of a decline. Even the most conservative model produced the same “strong and significant signal for a population bottleneck.”

They next used two computer-intensive statistical approaches to characterize the extent and timing of the decline, focusing on the two largest populations. The first statistical model (called the Beaumont method) makes specific demographic and mutation assumptions that can quantify the relative change in population size. Again, this model yielded the same result: orang-utan populations have declined by at least a factor of 50 (with 95% probability) or 100 (with 90% probability).

The second model (the Storz and Beaumont method) allowed the authors to estimate past and present population sizes and timing of the collapse. They found strong genetic evidence of a collapse for both Sabah populations, and a likely time frame within the past decades—coinciding with accelerated deforestation in the region in the 1950s and 1970s. This result also supports the 1987 estimate of 20,000 and the current estimate of 11,000 Sabah orang-utans—which means the decline was far more recent, and far more drastic, than previously assumed.

Normally, the genetic effects of such recent events would be obscured by ancient demographic events, such as those following major climatic changes, or the arrival of the first hunter-gatherers or farmers. But humans have so devastated orang-utan populations that the genetic signature of the recent bottleneck may have overshadowed any previous population fluctuations. These results demonstrate that genetic data can provide independent and extremely detailed information by detecting and quantifying the consequences of human impacts on endangered species. They also underscore the need to act now to protect the long-term survival of the species. The animals show enough genetic diversity to stabilize, the authors argue, if immediate steps are taken to reconnect remnant forest patches and halt further deforestation. Otherwise, humans will have restricted the fabled forest man to the realm of memory—or a life behind bars in a zoo.

